# Glycaemic control and antidiabetic therapy in patients with diabetes mellitus and chronic kidney disease – cross-sectional data from the German Chronic Kidney Disease (GCKD) cohort

**DOI:** 10.1186/s12882-016-0273-z

**Published:** 2016-06-11

**Authors:** Martin Busch, Jennifer Nadal, Matthias Schmid, Katharina Paul, Stephanie Titze, Silvia Hübner, Anna Köttgen, Ulla T. Schultheiss, Seema Baid-Agrawal, Johan Lorenzen, Georg Schlieper, Claudia Sommerer, Vera Krane, Robert Hilge, Jan T. Kielstein, Florian Kronenberg, Christoph Wanner, Kai-Uwe Eckardt, Gunter Wolf, on behalf of the GCKD Study Investigators, Georg Schlieper, Katharina Findeisen, Elfriede Arweiler, Sabine Ernst, Mario Unger, Jürgen Floege, Elke Schaeffner, Seema Baid-Agrawal, Kerstin Petzold, Ralf Schindler, Stephanie Titze, Karl F. Hilgers, Silvia Hübner, Susanne Avendano, Dinah Becker-Grosspitsch, Kai-Uwe Eckardt, Anna Köttgen, Ulla T. Schultheiss, Simone Meder, Erna Mitsch, Ursula Reinhard, Gerd Walz, Johan Lorenzen, Jan T. Kielstein, Petra Otto, Hermann Haller, Claudia Sommerer, Claudia Föllinger, Tanja Löschner, Martin Zeier, Martin Busch, Katharina Paul, Lisett Dittrich, Gunter Wolf, Thomas Sitter, Robert Hilge, Claudia Blank, Michael Fischereder, Vera Krane, Daniel Schmiedeke, Sebastian Toncar, Daniela Cavitt, Karina Schönowsky, Stefan Franz, Christoph Wanner, Kai-Uwe Eckardt, Stephanie Titze, Birgit Hausknecht, Marion Rittmeier, Anke Weigel, Hans-Ulrich Prokosch, Barbara Bärthlein, Kerstin Haberländer, Andreas Beck, Thomas Ganslandt, Sabine Knispel, Thomas Dressel, Olaf Gefeller, Martina Malzer, Jennifer Nadal, Matthias Schmid, André Reis, Arif B. Ekici, Florian Kronenberg, Barbara Kollerits, Hansi Weißensteiner, Lukas Forer, Sebastian Schönherr, Peter Oefner, Wolfram Gronwald

**Affiliations:** Department of Internal Medicine III, University Hospital Jena - Friedrich Schiller University, Erlanger Allee 101, D – 07747 Jena, Germany; Institute of Medical Biometry, Informatics and Epidemiology, University of Bonn, Bonn, Germany; Department of Nephrology and Hypertension, University of Erlangen-Nürnberg, Erlangen, Germany; Department of Internal Medicine IV, Medical Center University of Freiburg, Freiburg, Germany; Department of Medicine, Division of Nephrology and Medical Intensive Care, University Hospital Charité, Berlin, Germany; Hannover Medical School, Clinic for Nephrology, Hannover, Germany; Department of Medicine II - Nephrology and Clinical Immunology, University Hospital Aachen, Aachen, Germany; Department of Medicine, Division of Nephrology, University Hospital Heidelberg, Heidelberg, Germany; Department of Medicine I, Division of Nephrology, University Hospital Würzburg, Würzburg, Germany; Department of Medicine IV, Division of Nephrology, University Hospital of Ludwig-Maximilians University Munich, Munich, Germany; Division of Genetic Epidemiology, Medical University of Innsbruck, Innsbruck, Austria

**Keywords:** Chronic kidney disease, Diabetes mellitus, Glycaemic control, Hemoglobin A1C, Insulin therapy, Oral antidiabetic drugs

## Abstract

**Background:**

Diabetes mellitus (DM) is the leading cause of end-stage renal disease. Little is known about practice patterns of anti-diabetic therapy in the presence of chronic kidney disease (CKD) and correlates with glycaemic control. We therefore aimed to analyze current antidiabetic treatment and correlates of metabolic control in a large contemporary prospective cohort of patients with diabetes and CKD.

**Methods:**

The German Chronic Kidney Disease (GCKD) study enrolled 5217 patients aged 18–74 years with an estimated glomerular filtration rate (eGFR) between 30–60 mL/min/1.73 m^2^ or proteinuria >0.5 g/d. The use of diet prescription, oral anti-diabetic medication, and insulin was assessed at baseline. HbA1c, measured centrally, was the main outcome measure.

**Results:**

At baseline, DM was present in 1842 patients (35 %) and the median HbA1C was 7.0 % (25^th^–75^th^ percentile: 6.8–7.9 %), equalling 53 mmol/mol (51, 63); 24.2 % of patients received dietary treatment only, 25.5 % oral antidiabetic drugs but not insulin, 8.4 % oral antidiabetic drugs with insulin, and 41.8 % insulin alone. Metformin was used by 18.8 %. Factors associated with an HbA1C level >7.0 % (53 mmol/mol) were higher BMI (OR = 1.04 per increase of 1 kg/m^2^, 95 % CI 1.02–1.06), hemoglobin (OR = 1.11 per increase of 1 g/dL, 95 % CI 1.04–1.18), treatment with insulin alone (OR = 5.63, 95 % CI 4.26–7.45) or in combination with oral antidiabetic agents (OR = 4.23, 95 % CI 2.77–6.46) but not monotherapy with metformin, DPP-4 inhibitors, or glinides.

**Conclusions:**

Within the GCKD cohort of patients with CKD stage 3 or overt proteinuria, antidiabetic treatment patterns were highly variable with a remarkably high proportion of more than 50 % receiving insulin-based therapies. Metabolic control was overall satisfactory, but insulin use was associated with higher HbA1C levels.

**Electronic supplementary material:**

The online version of this article (doi:10.1186/s12882-016-0273-z) contains supplementary material, which is available to authorized users.

## Background

Chronic kidney disease (CKD) is a major complication of diabetes mellitus (DM) occurring in approximately one third of diabetic patients. DM is the leading cause for end-stage renal disease (ESRD) in most countries worldwide [1]. CKD of all etiologies potentiates cardiovascular disease (CVD) risk, depending on its severity [2]. The co-incidence of DM and CKD leads to a particularly marked increase in CVD risk [3]. Given the increasing prevalence of DM, the burden of diabetic kidney disease is expected to further increase in the future [4].

Good glycaemic control is the mainstay for preventing microvascular complications in patients with DM [5, 6]. Hemoglobin A1c (HbA1C), which reflects average glycaemic control over the past one to two months, has been shown to better capture increased risk for adverse events than plasma glucose [7]. Meta-analyses reported an increase of CVD events by approximately 18 % per increase of one percent of HbA1C [8]. HbA1C targets <7 % were shown to slow the progression of kidney disease [5, 9]. Intensive compared to standard glycaemic control also reduced coronary events in type 2 DM in some [10], albeit not all studies [11]. On the other hand the risk of severe hypoglycemia is doubled during intensive treatment, especially when the eGFR is <60 mL/min/1.73 m^2^ [12].

There are multiple therapeutic options for glycaemic control and they are likely to increase further with the introduction of new oral antidiabetic drugs. However, no clear recommendations exist for antidiabetic therapies in patients with moderately severe CKD and individualized treatment approaches and targets are so far recommended [13]. Recommendations of regulatory agencies, such as the FDA (Federal Drug Administration) for example for the use of metformin were recently challenged.

Understanding treatment practice and the factors associated with good and poor metabolic control appears important to assess the potential and limitations of different therapeutic regimes and to design future trials comparing different treatment regimes prospectively. Unfortunately, however, data about the use of different glucose-lowering strategies and their combinations in CKD patients are very sparse [14, 15]. We therefore decided to analyze a large prospective database from German kidney centers [16, 17], to understand how people with type 2 DM and CKD are currently treated and which factors are associated with metabolic control and with the use of the various classes of antidiabetic drugs.

## Methods

### Study population and design

The German Chronic Kidney Disease (GCKD) study is a prospective observational cohort study of patients with moderately severe CKD. It was approved by local ethics committees and registered in the national registry of clinical studies (DRKS 0003971). 5217 patients aged 18 to 74 years having either an estimated glomerular filtration rate (eGFR) between 30–60 mL/min/1.73 m^2^ (according to the 4-variable MDRD formula [18]) or proteinuria >0.5 g/day (or equivalent measures) were enrolled across nine German regional study centres after obtaining written informed consent. All patients are under regular care by nephrologists. Main criteria for exclusion from the GCKD study were non-Caucasian ethnicity, the presence of active malignancy, heart failure stage NYHA IV and any former transplantations. A summary of the baseline data was recently published [17]. Patients will be prospectively followed for up to 10 years. Details of the study design, enrolment process and study procedures have been reported [16].

During a structured interview at baseline each patient was asked about concomitant diseases including previous cardiovascular events; medications including antidiabetic drugs and dietary therapy for diabetes were recorded. Drugs were coded according to the Anatomical Therapeutic Chemical (ATC) classification. The treating nephrologists provided the patient’s medical history particularly of diabetes, kidney and CV diseases, and kidney biopsy reports.

### Definition of diabetes mellitus

For study purposes, DM was defined according to ADA criteria [19] when a HbA1C >6.5 % was present and/or if a patient was treated with any antidiabetic drug or antidiabetic diet.

### Laboratory analysis

At baseline, blood, plasma, serum and spot-urine samples were collected from each patient according to standard operation procedures, processed and shipped frozen to the central laboratory [16]. HbA1C and hemoglobin were determined from thawed whole blood. HbA1C was measured using an International Federation of Clinical Chemistry and Laboratory Medicine (IFCC) proven immunoassay (Cobas Integra 400 Plus, ROCHE Diagnostics, Switzerland). HbA1c results are reported in both NGSP (%) and IFCC (mmol/mol) units. According to recent recommendations, GFR was estimated using the CKD-EPI formula [20].

### Statistical methods

Baseline values of continuous variables are presented as mean ± standard deviation or median with 25^th^, 75^th^ percentiles. Values of categorical variables are presented as numbers and percentages. Spearman rank correlation coefficients were used to estimate correlation between continuous variables. Kruskal-Wallis tests were used to compare differences between independent groups of patients. In addition, Chi-Squared tests were used to evaluate associations between categorical variables. Effects of treatment on HbA1C levels were estimated by using stepwise logistic regression analysis with dependent variable “HbA1C below/above median of 7 %”. This dichotomization was chosen based on median calculation (see below) and is in accordance with clinical relevance [21, 22] and evidence reporting differences in outcomes in randomized controlled trials [10]. Using forward/backward stepwise selection as well as the inclusion of all covariates, models were adjusted for age, sex, body mass index (BMI), duration of CKD, physical activity, eGFR, hemoglobin, C-reactive protein, and antidiabetic medication (using dietary treatment alone as the reference category). A two-sided *p* value <0.05 was considered significant. Statistical analyses were performed with SAS 9.2 (SAS Institute, Inc., Cary, NC).

## Results

### Baseline characteristics

Diabetes was diagnosed in 1842 of the 5217 GCKD patients and differences between those with and without diabetes have been published recently [17]. In brief, patients with DM were significantly older than patients without DM (65 ± 8 vs. 58 ± 13 years, *p* < 0.001), and the proportion of male patients was higher (67 vs. 56 %), *p* < 0.001). Estimated GFR values were not significantly different (45 ± 16 mL/min/1.73 m^2^ in DM vs. 48 ± 17 mL/min/1.73 m^2^ in Non-DM, *p* = 0.07). The same was true for the urinary albumin/creatinine-ratio (UACR) (47 (9, 371) mg/g in DM vs. 54 (9, 397) mg/g in Non-DM, *p* = 0.45). In 213 patients with DM (12 %), eGFR was >60 mL/min/1.73 m^2^. Only 107 patients had type 1 diabetes (mean age 57.8 ± 11.3 years, 67 (63 %) male, median eGFR 45 mL/min/1.73 m^2^ (36, 56), UACR 72 mg/g (8, 307). The self-reported duration of DM was ≥5 years in 1046 patients (57 %), 1–5 years in 236 (13 %), and <1 year in 46 (2.5 %). In 514 patients the duration of DM was not known (28 %) and thus this factor was excluded from further statistical analysis. As main cause of CKD, the treating nephrologists listed diabetic nephropathy in 40 %, vascular nephropathy in 17 %, glomerulonephritis in 8 %, interstitial nephritis in 3 %, systemic disease in 3 %, and miscellaneous in 29 %. The rate of self-reported diabetic retinopathy was 20 % (*n* = 376).

The median HbA1C value of the study cohort was 7.0 % (6.8, 7.9), 53 mmol/mol (51, 63). Clinical data of patients with DM stratified according to their baseline HbA1C (equal or below vs above median) are shown in Table 1. Most characteristics were similar between both groups, except that patients with an HbA1C ≤7.0 % were on average one year older, had slightly lower systolic blood pressure and BMI, lower UACR and CRP.

**Table 1:** Baseline data of 1842 patients with diabetes mellitus and CKD stratified by median HbA1C levels (7.0 %, 53 mmol/mol)

	HbA1C ≤ 7.0 % (53 mmol/mol) *n =* 897	HbA1C > 7.0 % (53 mmol/mol) *n* = 945
Epidemiological data		
Age (years)	65 ± 8	64 ± 8
Male, number (%)	591 (65.9)	637 (67.4)
Systolic blood pressure (mm Hg)	141 ± 22	143 ± 21
Diastolic blood pressure (mm Hg)	76 ± 12	76 ± 12
BMI (kg/m^2^)	32 ± 6	33 ± 6
Current smokers, number (%)	127 (14.2)	142 (15.0)
Duration of CKD		
≥ 5 years	369 (41.2)	413 (43.7)
3 – < 5 years	133 (14.8)	162 (17.1)
1 – < 3 years	207 (23.1)	198 (21.0)
< 1 year	154 (17.2)	130 (13.8)
Physical activity		
Less than once a week	173 (19.3)	201 (21.3)
1–2 times a week	202 (22.6)	221 (23.4)
3–5 times a week	256 (28.6)	236 (25.0)
More than 5 times a week	251 (28.0)	278 (29.5)
CV disease, number (%) ^a^	403 (44.9)	463 (49.0)
Laboratory data		
Creatinine (mg/dL)	1.47 (1.23, 1.80)	1.50 (1.27, 1.83)
eGFR (mL/min/1.73 m^2^)	44 (35, 55)	44 (35, 54)
eGFR 0–29	126 (14.1)	125 (13.6)
eGFR 30–44	322 (36.1)	346 (37.6)
eGFR 45–59	296 (33.2)	301 (32.7)
eGFR ≥ 60	148 (16.6)	149 (16.2)
Urinary albumin/creatinine-ratio (mg/gCrea)	34 (7, 330)	59 (10, 412)
< 30	420 (48.4)	356 (38.7)
30–300	216 (24.9)	298 (32.4)
> 300	231 (26.6)	266 (28.9)
Hemoglobin (g/dL) ^a^	13.4 (12.1, 14.5)	13.6 (12.5, 14.5)
HbA1C (%)	6.6 (6.3, 6.8)	7.9 (7.4, 8.6)
HbA1C (mmol/mol)	49 (45, 51)	63 (57, 70)
Serum albumin (g/L)	38.6 (35.9, 40.6)	38.1 (35.6, 40.5)
CRP (mg/L)	2.50 (1.24, 5.78)	3.13 (1.47, 6.73)
Calcium (mmol/L)	2.27 (2.18, 2.35)	2.28 (2.19, 2.36)
Phosphate (mmol/L)	1.11 (0.97, 1.25)	1.09 (0.96, 1.23)
Ca/Ph-Produkt (mmol^2^/L^2^)	2.03 (1.79, 2.33)	2.06 (1.79, 2.33)
Total cholesterol (mg/dL) ^b^	194.9 (167.4, 228.8)	192.2 (161.2, 223.7)
HDL (mg/dL) ^b^	45.1 (37.4, 55.7)	43.2 (35.8, 53.4)
LDL (mg/dL) ^b^	103.1 (82.9, 131.8)	99.4 (75.3, 124.3)
TG (mg/dl) ^c^	180.9 (125.5, 259.8)	197.1 (134.8, 279.4)
Antidiabetic treatment strategies		
Dietary treatment alone	292 (35.1)	113 (13.5)
Oral anti-diabetic drugs alone, *any*	277 (33.3)	149 (17.8)
Oral anti-diabetic drugs plus insulin	49 (5.9)	92 (10.9)
Insulin alone	214 (25.7)	485 (57.8)

Values are reported as mean values ± standard deviation, medians (25^th^, 75^th^ percentile), or numbers (percentages), as appropiate. ^a^ The composite of cardiovascular disease includes all patients with one or more of the following: cardiac valve replacement, aortic aneurysm, coronary artery disease, cerebrovascular disease, peripheral artery disease

^a^ For conversion into SI units (mmol/L): multiply with 0.62

^b^ For conversion into SI units (mmol/L): multiply with 0.02586

^c^ For conversion into SI units (mmol/L): multiply with 0.0114

### Antidiabetic treatment

Roughly one quarter of the patients with DM were treated with an antidiabetic diet regimen only (24.2 %), or received oral antidiabetic drugs, but no insulin (25.5 %). The majority was treated with insulin only (41.8 %) and a small group was on insulin and oral antidiabetic agents (8.4 %) (Table 2). Differences across groups of different therapeutic strategies including classes of oral antidiabetic drugs, alone or in combination, are presented in Table 2. Patients who were treated with insulin alone were significantly younger but exhibited more advanced kidney disease with a lower eGFR and higher UACR. Moreover, they had the highest rate of pre-existing CVD; 88 out of the 699 patients in this group had type 1 diabetes. The opposite was true for patients being treated with oral antidiabetic drugs but not insulin. These patients had the highest eGFR, the lowest UACR, and the lowest rate of CVD. Their HbA1C was significantly lower (6.7 % (6.3, 7.3), 50 mmol/mol (45, 56)) as compared to the groups being treated with insulin, either alone (7.5 % (6.8, 8.4), 58 mmol/mol (51, 68)), or in the combination with oral antidiabetic drugs (7.5 % (6.8, 8.4), 58 mmol/mol (51, 68), *p* < 0.0001 resp.). Almost one fifth of patients (18.8 %) received metformin alone or in any combination. In this group, eGFR was significantly higher as compared to patients not using metformin (53 (43, 62) vs. 42 (34, 52) mL/min/1.73 m^2^, *p* < 0.0001). The lowest UACR (29 mg/g (6, 202)) was observed in those treated with DPP-4 inhibitors, alone or in combination. Only a small minority of 2.4 % (*n* = 45) were treated by GLP-1-agonists, either alone or in different combinations and were thus excluded from further statistical analysis.

**Table 2:** Patient characteristics in patients with diabetes mellitus and CKD across different antidiabetic treatment regimens

	Dietary treatment	Oral anti-diabetic drugs alone, *any*	Oral anti-diabetic drugs plus insulin	Insulin alone	*P*-value ^a^	Classes of oral antidiabetic drugs, alone or in combination			
						Metformin	Sulfonyl-ureas	Glinides	DPP-4-inhibitors
Number	405	426	141	699		346	265	119	191
Percent	24.2	25.5	8.4	41.8		18.8	14.4	6.5	10.4
Age, *years*	65 ± 8	65 ± 7	65 ± 7	64 ± 9	0.0377	64 ± 8	67 ± 6	66 ± 6	65 ± 7
Male, *n (%)*	270 (66.7)	282 (66.2)	89 (63.1)	478 (68.4)	0.64	213 (61.6)	175 (66.0)	92 (77.3)	129 (67.5)
BMI, *kg/m* ^*2*^	31 ± 6	32 ± 6	35 ± 6	32 ± 6	<0.0001	33 ± 6	33 ± 6	32 ± 6	34 ± 6
Hemoglobin, *g/dL* ^*c*^	13.6 (12.5, 14.8)	13.5 (12.2, 14.5)	13.6 (12.6, 14.5)	13.3 (12.2, 14.4)	0.0833	13.6 (12.3, 14.7)	13.4 (12.5, 14.5)	13.5 (12.5, 14.6)	13.6 (12.5, 14.6)
HbA1c, *%*	6.7 (6.5, 7.1)	6.7 (6.3, 7.3)	7.5 (6.8, 8.4)	7.5 (6.8, 8.4)	<0.0001	6.9 (6.4, 7.6)	7.1 (6.5, 7.9)	6.8 (6.5, 7.6)	6.9 (6.4, 7.7)
HbA1c, *mmol/mol*	50 (48, 54)	50 (45, 56)	58 (51, 68)	58 (51, 68)		52 (46, 60)	54 (48, 63)	51 (48, 60)	52 (46, 61)
eGFR, *mL/min/1.73 m* ^*2*^	44 (35, 55)	48 (38, 58)	46 (37, 56)	41 (33, 51)	<0.0001	53 (43, 62)	45 (36, 55)	42 (33, 50)	47 (36,56)
UACR, *mg/gCrea*	34 (8, 253)	31 (8, 322)	52 (9, 333)	71 (11, 512)	0.0011	38 (7, 369)	50 (9, 329)	45 (9, 371)	29 (6, 202)
CV disease, *n(%)* ^*b*^	176 (43.5)	169 (39.7)	72 (51.1)	382 (54.7)	<0.0001	141 (40.7)	120 (45.3)	46 (38.7)	88 (46.1)
Duration of CKD									
≥ 5 years	183 (45.3)	152 (35.7)	56 (39.7)	324 (46.4)	0.0021	121 (34.9)	99 (37.4)	50 (42.0)	68 (35.6)
3 – < 5 years	65 (16.1)	63 (14.8)	23 (16.3)	115 (16.5)		50 (14.5)	35 (13.2)	20 (16.8)	28 (14.7)
1 – < 3 years	80 (19.8)	108 (25.3)	25 (17.7)	152 (21.8)		81 (23.4)	69 (26.0)	30 (25.2)	47 (24.6)
< 1 year	63 (15.6)	84 (19.7)	22 (15.6)	84 (12)		66 (19.1)	50 (18.9)	17 (14.3)	41 (21.4)

Values are reported as numbers and percentages (based on all diabetic patients), mean values ± standard deviation, or medians (25^th^, 75^th^ percentile), as appropiate

Missings (*n* = 171) resulted mostly from combinations that were used less frequently

^a^ according to Kruskal-Walis test, analysis of variance, or Chi-Square test, as appropriate

^b^ The composite of cardiovascular disease includes all patients with one or more of the following: cardiac valve replacement, aortic aneurysm, coronary artery disease, cerebrovascular disease, peripheral artery disease

^c^ For conversion into SI units (mmol/L): multiply with 0.62

The eleven most commonly used antidiabetic regimes, based on individual drugs and the corresponding clinical data are presented in Table 3. The metformin alone group showed the lowest HbA1C of 6.6 % (6.3, 7.1), 49 mmol/mol (45, 54) and the highest eGFR (56 ± 18 mL/min/1.73 m^2^) in combination with the lowest UACR (22 mg/gCrea (5, 247)). The highest levels of HbA1C were found in the small number of patients treated with insulin and sulfonylureas (7.8 % (7.0, 8.6), 62 mmol/mol (53, 70)) followed by patients who were treated with insulin and DPP-4 inhibitors having an HbA1C of 7.6 % (6.6, 8.7), 60 mmol/mol (47, 72). These two groups had the lowest average eGFR levels (42 ± 12 and 41 ± 12 mL/min/1.73 m^2^, respectively).

**Table 3:** Patient characteristics according to treatment with the 11 most commonly used antidiabetic treatment strategies in 1842 patients with diabetes mellitus and CKD

	Insulin	Dietary treatment	Metformin	Sulfonyl-ureas	Metformin + Insulin	Glinides	DPP-4 inhibitors	Metformin + Sulfonyl-ureas	Sulfonyl-ureas + Insulin	Metformin + DPP-4 inhibitors	DPP-4 inhibitors + Insulin
N	699	405	123	123	76	59	46	38	38	37	27
Percent	41.8	24.2	7.4	7.4	4.6	3.5	2.8	2.3	2.3	2.2	1.6
Age, *years*	64 ± 9	65 ± 8	64 ± 9	67 ± 6	64 ± 7	66 ± 6	65 ± 7	66 ± 5	66 ± 6	64 ± 8	65 ± 7
Male gender, *n (%)*	478 (68.4)	270 (66.7)	70 (56.9)	80 (65.0)	46 (60.5)	45 (76.3)	36 (78.3)	29 (76.3)	27 (71.1)	22 (59.5)	16 (59.3)
BMI, *kg/m* ^*2*^	32 ± 6	31 ± 6	33 ± 6	32 ± 5	35 ± 5	31 ± 5	34 ± 5	33 ± 6	34 ± 8	32 ± 5	36 ± 6
Hemoglobin, *g/dL* ^b^	13.2 (12.2, 14.4)	13.6 (12.5, 14.8)	13.7 (12.2, 5.0)	13.4 (12.3, 14.5)	13.7 (12.4, 14.4)	13.5 (12.4, 14.5)	13.1 (12.1, 14.3)	13.1 (12.1, 14.4)	13.7 (12.7, 14.7)	14.1 (12.5, 15.1)	13.5 (12.8, 14.1)
HbA1c, *%* ^c^	7.5 (6.8, 8.4)	6.7 (6.5, 7.1)	6.6 (6.3, 7.1)	6.8 (6.3, 7.5)	7.3 (6.8, 8.1)	6.7 (6.3, 7.0)	6.7 (6.3, 7.1)	7.3 (6.6, 7.9)	7.8 (7.0, 8.6)	6.7 (6.4, 7.2)	7.6 (6.6, 8.7)
eGFR, *mL/min/1.73 m* ^*2*^	43 ± 15	46 ± 17	56 ± 18	45 ± 12	55 ± 17	42 ± 10	45 ± 13	52 ± 16	42 ± 12	53 ± 17	41 ± 12
UACR, *mg/gCrea*	71 (11, 512)	34 (8, 253)	22 (7, 322)	45 (8, 319)	59 (7, 353)	51 (8, 245)	22 (5, 247)	56 (14, 408)	81 (12, 357)	29 (6, 441)	29 (14, 102)
Duration of CKD, *n (%)*											
≥ 5 years	324 (46.4)	183 (45.3)	38 (30.9)	47 (38.2)	32 (42.1)	27 (45.8)	16 (34.8)	10 (26.3)	14 (36.8)	14 (37.8)	10 (37)
3 – < 5 years	115 (16.5)	65 (16.1)	19 (15.5)	17 (13.8)	13 (17.1)	10 (17)	9 (19.6)	6 (15.8)	4 (10.5)	2 (5.4)	6 (22.2)
1 – < 3 years	152 (21.8)	80 (19.8)	29 (23.6)	33 (26.8)	11 (14.5)	15 (25.4)	9 (19.6)	11 (29)	10 (26.3)	11 (29.7)	4 (14.8)
< 1 year	84 (12)	63 (15.6)	27 (22)	25 (20.3)	11 (14.5)	6 (10.2)	11 (23.9)	7 (18.4)	5 (13.2)	8 (21.6)	6 (22.2)
CV disease, *number (%)* ^*a*^	382 (54.7)	176 (43.5)	38 (30.9)	58 (47.2)	38 (50)	17 (28.8)	17 (37)	23 (60.5)	16 (42.1)	16 (43.2)	18 (66.7)

Values are reported as numbers and percentages (based on all diabetic patients), mean values ± standard deviation, or medians (25^th^, 75^th^ percentile), as appropriate

Missings (*n* = 171) resulted mostly from combinations that were used less frequently

^a^ The composite of cardiovascular disease includes all patients with one or more of the following: cardiac valve replacement, aortic aneurysm, coronary artery disease, cerebrovascular disease, peripheral artery disease

^b^ For conversion of hemoglobin into SI units (mmol/L): multiply with 0.62

^c^ For conversion of HbA1C into IFCC units (mmol/mol): (10.93 × HbA1C in %)-23.5

### Glycaemic control and hemoglobin

**Fig. 1:**
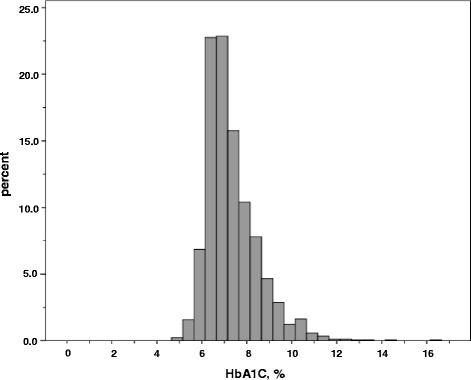
Histogram of observed hemoglobin A1C (HbA1C) values in 1842 patients with diabetes mellitus and stage 3 CKD and/or overt proteinuria, for conversion of HbA1C into IFCC units (mmol/mol): (10.93 × HbA1C in %)-23.5

The distribution of the HbA1C values is shown in Fig. 1. A great number of patients cluster around the median of 7.0 % (53 mmol/mol). Fourtyfive percent of the patients had HbA1C values within a range of 6.5–7.5 % (48–58 mmol/mol), 20 % below 6.5 %, and 35 % ≥7.5 %. There was a slight positive correlation of hemoglobin with HbA1C (*r* = 0.082, *p* = 0.001, Additional file 1: Figure S1) and eGFR (*r* = 0.247, *p* < 0.0001, Additional file 2: Figure S2). No significant association of HbA1C with eGFR was found. Median HbA1c among the 107 patients with type 1 DM was 7.9 % (7.1, 8.8), 63 mmol/mol (54, 73), which was significantly higher compared with patients having type 2 DM (*n* = 1678, HbA1C 7.0 % (6.5, 7.8), 53 mmol/mol (48, 62), *p* < 0.0001).

### Factors associated with HbA1C

Factors associated with an HbA1C level above the median of 7.0 % (53 mmol/mol) were determined. For this analysis, clinical parameters (see method section) and different treatment strategies (oral and/or insulin treatment, dietary treatment as reference, see Table 2) were included. Factors significantly associated with an HbA1C level >7.0 % were higher body mass index (OR 1.038, *p* < 0.0001) and higher hemoglobin (OR 1.112, *p* = 0.001, Table 4). The use of oral antidiabetic drugs alone was not significantly associated with the probability of a median HbA1C >7.0 % (Table 4). Excluding the use of sulfonylureas did not increase the probability of an HbA1C >7.0 % in those receiving oral anti-diabetic drugs only (OR 0.898, 95 % CI 0.625–1.291, *p* = 0.56). The use of insulin, either alone (OR 5.634, *p* < 0.0001) or in the combination with oral antidiabetic drugs (OR 4.233, *p* < 0.0001), was significantly associated with median HbA1C levels >7.0 %. The entire model is presented in Additional file 3: Table S1.

**Table 4:** Correlates of median HbA1C levels >7.0 % (53 mmol/mol) according to stepwise logistic regression analysis (final model)

Indicators ^a, b^	Regression coefficient ^a^	Standard error ^a^	Odds ratio ^a^	95 % confidence interval ^a^	*P*-value ^a^
Body mass index (per 1 kg/m^2^ increase)	0.0374	0.0091	1.038	1.020–1.057	<0.0001
Hemoglobin (per 1 g/dL increase) ^c^	0.1060	0.0323	1.112	1.044–1.184	0.0010
Antidiabetic treatment strategies ^b^					
Oral anti-diabetic drugs alone, *any* (*n* = 426)	0.2177	0.1566	1.243	0.915–1.690	0.16
Oral anti-diabetic drugs plus insulin (*n* = 141)	1.4428	0.2159	4.233	2.772–6.462	<0.0001
Insulin alone (*n* = 699)	1.7288	0.1427	5.634	4.260–7.452	<0.0001

*N* = 266 observations were excluded from the analysis due to combinations that were used less frequently and missing values

^a^ According to the final model; age, gender, BMI, duration of CKD, physical activity, eGFR, hemoglobin, CRP, and antidiabetic medication were used as variables in the initial model

^b^ Dietary treatment was used as the reference category for any group of antidiabetic therapy

^c^ For conversion into SI units (mmol/L): multiply with 0.62

We further analyzed the association of different treatment groups as outlined in Table 3 with HbA1C: The use of insulin, either alone or in combination with sulfonylureas, metformin, or DPP-4 inhibitors was significantly associated with median HbA1C levels >7.0 % (OR between 3.373 and 7.726, *p* < 0.0001, Additional file 4: Table S2). In contrast, the monotherapy with oral antidiabetic drugs such as metformin (OR 0.895, *p* = 0.0002), DPP-4 inhibitors (OR 0.864, *p* = 0.0117), or glinides (OR 0.898, *p* = 0.0069), and the combination of metformin with DPP-4 inhibitors (OR 0.970, *p* = 0.0417) was significantly associated with a decreased probability of median HbA1C levels >7.0 %. Instead, treatment with sulfonylureas, either alone (OR 1.636, *p* = 0.31) or in the combination with metformin (OR 3.497, *p* = 0.07) or insulin (OR 7.726, *p* = 0.0002) was associated with an increased probability of median HbA1C levels >7.0 % (Additional file 4: Table S2). The entire model is given in Additional file 5: Table S3.

## Discussion

This study describes antidiabetic treatment reality in a large cohort of CKD patients with DM. All patients were under routine care of nephrologists and some of them were additionally seen by diabetologists, so that the data have to be interpreted as refecting specialist care.

A major finding of the analysis is that given the median HbA1C of 7.0 % (53 mmol/mol) [21, 22], the overall quality of DM control appears to be satisfactory in most of the patients despite the combination of CKD and DM. The treatment quality is comparable or even better than in large cohort studies of people with type 2 diabetes in Germany that have found mean HbA1C values of 7.0 and 7.2 % [23, 24]. German guidelines recommend an HbA1C between 6.5 and 7.5 % (48–58 mmol/mol) for all patients with DM irrespective of concomitant kidney disease [22] and 45 % of our cohort met this criterium. The current U.S. National Kidney Foundation’s Kidney Disease Outcome Initiative (K/DOQI) guidelines recommend a target HbA1C “*of ~7.0 % to prevent or delay progression of the microvascular complications of DM, including diabetic kidney disease*” [21]. Thus, many of our patients were treated according to these guidelines. Other studies also confirm that good metabolic control can be achieved in patients with DM and CKD. In a Canadian population based study with 23,296 participants with DM and an eGFR of lower than 60 mL/min/1.73 m^2^, but not on dialysis, a median HbA1C level of 6.9 % was found [25]. This study also reported an increase in the risk of mortality at HbA1C levels of >8.0 and <6.5 % [25]. In this regard, it is noteworthy that 20 % of our cohort had an HbA1C below 6.5 % (48 mmol/mol), which might even suggest overtreatment or poor health status with an increased risk of hypoglycemia [26]. On the other hand 24.7 % of patients with DM and CKD in the GCKD study had an HbA1C >8.0 % (64 mmol/mol), indicating relevant heterogeneity and opportunities for improvement.

Interestingly the median dichotomization of our total cohort with a median HbA1C of 6.6 % (49 mmol/mol) in the lower group and a median of 7.9 % (63 mmol/mol) in the higher group corresponds to an intensive versus standard treatment approach when compared with the mean HbA1C values of the intensive (6.7 %, 50 mmol/mol) and standard (7.7 %, 61 mmol/mol) treatment groups in the ADVANCE, ACCORD, and the VADT trial [10]. Although intensive compared with conventional glycaemic control did not result in significant differences for all-cause and CVD mortality, the risk of microvascular complications including kidney disease was lower in more intensively treated patients in these interventional trials [27, 28]. While our patients already had CKD at enrollment, it is possible that improved metabolic control retards the progression of already existing CKD. This may also reduce CVD morbidity and mortality in the long-term because any progression of CKD is associated with an exponential increase in CVD risk [2, 29].

CKD can be associated with anemia, which may limit the utility of HbA1c for diagnosing DM and assessing glycaemic control; HbA1C levels tend to be lower if renal anemia is present, due to a shortened life span of erythrocytes [30]. Indeed, we found a slight but significant positive correlation between hemoglobin and HbA1C. However, in the majority of patients hemoglobin values were in the normal range and there was no difference in the mean hemoglobin concentrations between patients below and above the median HbA1C value. In addition, hemoglobin but not HbA1C was positively correlated with eGFR, confirming previous findings [31].

A further important finding of our study is that antidiabetic treatment patterns differed from the general diabetes population and were overall highly variable. A total of slightly more than 50 % was treated with insulin-based therapies. In a German general type 2 diabetes population the portion of insulin-based therapies was 31 % which is 20 % lower than in the patients currently studied [32]. More than 40 % of our patients were treated with insulin alone which is distinctly higher as in general diabetes cohorts including German cohorts in which only 10–20 % are treated with insulin monotherapy [24, 33, 34]. Only one third of our patients was treated with oral glucose-lowering medication with or without insulin. In the German general diabetes population at least 60 to 70 % of the patients receive any oral antidiabetic medication [23, 24, 32]. One quarter of our cohort was treated with oral antidiabetic agents alone comparing with up to 75 % in general diabetes cohorts [24, 35]. However, this portion is apparently lower in German diabetes cohorts; at about 40 to a maximum of 60 % [23, 24]. Different treatments were associated with different levels of metabolic control. The use of insulin, alone or in the combination with oral antidiabetic drugs, was accompanied by a 4 to nearly 6 times higher probability of having HbA1C values >7.0 % (53 mmol/mol). Conversely, the use of oral glucose-lowering drugs alone, namely metformin, glinides, DPP-4 inhibitors, or the combination of metformin with DPP-4 inhibitors was not associated with such probability except sulfonylureas. The observational nature of the study precludes drawing conclusions on cause and effect when considering these differences in metabolic control in patients receiving different therapies and a number of factors may play a role. Thus it is not unlikely that patients whose diabetes was difficult to treat were switched to sulfonylureas or insulin, explaining at least in part higher HbA1C levels in these patients. On the other hand higher HbA1C targets may intentionally have been chosen in some patients. The K/DOQI guideline for CKD patients recommends that in patients “*with co-morbidities or limited life expectancy and risk of hypoglycemia*”, target HbA1C should be extended above 7.0 % [21]. While implementation of this recommendation could explain higher HbA1C levels in some patients, it appears unlikely that this applies to the majority of the insulin treated patients, given their younger age, no difference in eGFR, and only a slightly higher rate in prevalent CVD. On the other hand, their UACR was higher, the duration of CKD longer, and their physical activity was lower indicating more advanced diabetic disease. Furthermore, the K/DOQI guidelines recommend a HbA1C treatment target of >7.0 % for patients at risk of hypoglycemia, “*including those treated with insulins or sulfonylureas and/or have advanced CKD*” [21]. The amplified risk for hypoglycaemia in CKD is well documented, especially for patients treated with insulin or sulfonylureas [5]. Indeed the mean HbA1C levels in patients receiving sulfonylureas in combination with metformin or insulin were higher than in many other groups, but this did not apply to those treated with sulfonylureas only. Conversely, the presence of a lower HbA1C in the orally treated patients (except those having sulfonylureas) is unlikely due to more frequent episodes of hypoglycaemia as metformin and DPP-4 inhibitors do not cause hypoglycaemia and the risk for hypoglycaemia is very low with the use of glinides, especially in CKD stages lower than stage G4 [21]. Although patients difficult to treat with oral antidiabetic agents may have been switched to insulin, it is nevertheless noteworthy that a substantial proportion of patients was well controlled on oral agents only, indicating their potential value in the presence of CKD. Apart from the different antidiabetic therapies any increase in BMI was also significantly related to an HbA1C of >7.0 % (53 mmol/mol) and may point towards a subgroup of patients whose DM is more difficult to treat due to increased insulin resistance.

Irrespective of the underlying reasons the association of insulin use with worse metabolic control has previously also been observed in other patient populations. A very large retrospective analysis compared patients with type 2 DM aged 50 years and older, in whom treatment was escalated from oral monotherapy to either a combination therapy of different oral antidiabetic drugs or to a regimen that included insulin. The use of insulin treatment was associated with an increase in HbA1C (8.3 % versus 7.7 % in the oral combination group), increased mortality (HR 1.49), and the increased likelihood of a first large-vessel disease event [36]. In another retrospective study in type 2 DM patients from Germany, patients who were prescribed insulin or sulfonylurea, any combination of insulin with oral antidiabetic drugs, or the combination of sulfonylurea with metformin were least likely to achieve an intensive HbA1C target [34].

Another confounding influence is based on the fact that drug licenses constrain the prescription depending on the level of kidney function. This is of particular relevance for metformin, which at the time of study enrollment and until recently was not approved in Germany for patients with an eGFR below 60 mL/min/1.73 m^2^ (now changed to below 45 ml/min/1.73 m^2^). Accordingly the eGFR was higher in those receiving metformin and the overall rate of metformin use in our cohort was <20 %. This is distinctly lower as compared with current data from the U.S. National Health and Nutrition Examination Survey (NHANES) in which the rates of metformin use were 48.6 % and 57.4 % in eGFR ranges of 30 to 45 and >45 to 60 mL/min/1.73 m^2^ [37]. It should be noted that there is an ongoing debate whether the current thresholds for metformin use as suggested by guidelines may be too restrictive [38]. The American Diabetes Association and the European Association for the Study of Diabetes stated that the England National Clinical Guideline for Management in Primary and Secondary Care from the National Institute for Health and Care Excellence (NICE) [39] is more evidence based, generally allowing metformin use down to an eGFR of 30 mL/min/1.73 m^2^, with dose reduction advised at 45 mL/min/1.73 m^2^ [38–40]. This European guideline from 2009 may have prompted the German doctors as well, to prescribe metformin despite an eGFR of below 60 mL/min/1.73 m^2^.

Apart from its observational nature, there are further limitations of our study. We have no information on treatment duration and medication history, which would have allowed a better understanding on why patients were on particular therapies. In this regard there exists the possibility that patients on insulin are presumably the ones with more advanced disease with its attendant higher risk of complications and higher HbA1C. As a consequence, the treating physicians may have tended to use insulin in patients with a higher HbA1C and with complications when compared to patients with lower HbA1C. Thus, a causal association between the use of insulin and higher HbA1C and complications cannot be proven. Nevertheless, the cross-sectional nature of this baseline analysis prohibits conclusions on associations with outcomes. Although HbA1C still remains the cornerstone for the estimation of glycaemic control and as most clinical trials have used it [41], it is sensitive to episodes of hypoglycaemia.

The strength of the study includes its size and the assessment of different types of antidiabetic medication. Medication information was directly obtained from the patient, possibly overcoming some of the uncertainties of implementation and validity of prescription orders in patients treated by more than one physician. HbA1C values were all determined in a central lab using identical methodology and interference from carbamylated hemoglobin could be excluded by using a specific immunoassay [42].

## Conclusions

Within a large cohort of referred patients with DM and CKD stage 3 and/or overt proteinuria, the overall treatment quality of DM was satisfactory, but relevant proportions of patients had HbA1C values below or above the recommended target range. The underlying treatment patterns differed from general diabetes cohorts with a remarkably high proportion of more than 50 % receiving insulin-based therapies which were associated with an increased probability of HbA1C levels >7 % (53 mmol/mol). Future follow-up will reveal whether the level of control of DM in the presence of CKD and/or the choice of antidiabetic agent/s is associated with renal and CVD outcomes and differences in mortality.

## Abbreviations

CKD, chronic kidney disease; CRP, C-reactive protein; CV, cardiovascular; CVD, cardiovascular disease; DM, diabetes mellitus; eGFR, estimated glomerular filtration rate; ESRD, end-stage renal disease; GCKD, German Chronic Kidney Disease Study; K/DOQI, Kidney Disease Outcome Quality Initiative; NHANES, National Health and Nutrition Examination Survey; NYHA, New York Heart Association; UACR, urinary albumin/creatinine ratio

## Additional files

Additional file 1: Figure S1. Association between hemoglobin and hemoglobin A1C (HbA1C) values in 1775 patients having diabetes mellitus and stage 3 chronic kidney disease and/or overt proteinuria (*r* = 0.082, *p* < 0.001), for conversion of hemoglobin into SI units (mmol/L): multiply with 0.62. (PPT 81 kb) Additional file 2: Figure S2. Association between hemoglobin and estimated glomerular filtration rate (eGFR) in 1759 patients having diabetes mellitus and stage 3 chronic kidney disease and/or overt proteinuria (*r* = 0.247, *p* < 0.0001). (PPT 103 kb) Additional file 3: Table S1. Correlates of median HbA1C levels >7.0 % (53 mmol/mol) according to logistic regression analysis (entire model). (DOCX 24 kb) Additional file 4: Table S2. Correlates of median HbA1C levels >7.0 % (53 mmol/mol) including the most frequently used antidiabetic therapies according to stepwise logistic regression analysis (final model). (DOCX 22 kb) Additional file 5: Table S3. Correlates of median HbA1C levels >7.0 % (53 mmol/mol) including the most frequently used antidiabetic therapies according to logistic regression analysis (entire model). (DOCX 24 kb)

## References

[1] Jha V, Garcia-Garcia G, Iseki K, Li Z, Naicker S, Plattner B (2013). Chronic kidney disease: global dimension and perspectives. Lancet.

[2] Go AS, Chertow GM, Fan D, McCulloch CE, Hsu CY (2004). Chronic kidney disease and the risks of death, cardiovascular events, and hospitalization. N Engl J Med.

[3] Tonelli M, Muntner P, Lloyd A, Manns BJ, Klarenbach S, Pannu N (2012). Risk of coronary events in people with chronic kidney disease compared with those with diabetes: a population-level cohort study. Lancet.

[4] Wild S, Roglic G, Green A, Sicree R, King H (2004). Global prevalence of diabetes: estimates for the year 2000 and projections for 2030. Diabetes Care.

[5] UK Prospective Diabetes Study (UKPDS) Group (1998). Intensive blood-glucose control with sulphonylureas or insulin compared with conventional treatment and risk of complications in patients with type 2 diabetes (UKPDS 33). Lancet.

[6] Stratton IM, Adler AI, Neil HA, Matthews DR, Manley SE, Cull CA (2000). Association of glycaemia with macrovascular and microvascular complications of type 2 diabetes (UKPDS 35): prospective observational study. BMJ.

[7] Selvin E, Steffes MW, Zhu H, Matsushita K, Wagenknecht L, Pankow J (2010). Glycated hemoglobin, diabetes, and cardiovascular risk in nondiabetic adults. N Engl J Med.

[8] Selvin E, Marinopoulos S, Berkenblit G, Rami T, Brancati FL, Powe NR (2004). Meta-analysis: glycosylated hemoglobin and cardiovascular disease in diabetes mellitus. Ann Intern Med.

[9] The Diabetes Control and Complications Trial (DCCT) Research Group (1993). The effect of intensive treatment of diabetes on the development and progression of long-term complications in insulin-dependent diabetes mellitus. N Engl J Med.

[10] Ray KK, Seshasai SR, Wijesuriya S, Sivakumaran R, Nethercott S, Preiss D (2009). Effect of intensive control of glucose on cardiovascular outcomes and death in patients with diabetes mellitus: a meta-analysis of randomised controlled trials. Lancet.

[11] Hemmingsen B, Lund SS, Gluud C, Vaag A, Almdal T, Hemmingsen C (2011). Intensive glycaemic control for patients with type 2 diabetes: systematic review with meta-analysis and trial sequential analysis of randomised clinical trials. BMJ.

[12] Tuttle KR, Bakris GL, Bilous RW, Chiang JL, de Boer IH, Goldstein-Fuchs J (2014). Diabetic kidney disease: a report from an ADA Consensus Conference. Am J Kidney Dis.

[13] Inzucchi SE, Bergenstal RM, Buse JB, Diamant M, Ferrannini E, Nauck M (2015). Management of hyperglycaemia in type 2 diabetes, 2015: a patient-centred approach. Update to a position statement of the American Diabetes Association and the European Association for the Study of Diabetes. Diabetologia.

[14] del Pozo-Fernandez C, Pardo-Ruiz C, Sanchez-Botella C, Blanes-Castaner V, Lopez-Menchero R, Gisbert-Selles C (2012). Discrepancies among consensus documents, guidelines, clinical practice and the legal framework for the treatment of type 2 diabetes mellitus patients. Nefrologia.

[15] Solini A, Penno G, Bonora E, Fondelli C, Orsi E, Trevisan R (2013). Age, renal dysfunction, cardiovascular disease, and antihyperglycemic treatment in type 2 diabetes mellitus: findings from the Renal Insufficiency and Cardiovascular Events Italian Multicenter Study. J Am Geriatr Soc.

[16] Eckardt KU, Barthlein B, Baid-Agrawal S, Beck A, Busch M, Eitner F (2012). The German Chronic Kidney Disease (GCKD) study: design and methods. Nephrol Dial Transplant.

[17] Titze S, Schmid M, Kottgen A, Busch M, Floege J, Wanner C (2015). Disease burden and risk profile in referred patients with moderate chronic kidney disease: composition of the German Chronic Kidney Disease (GCKD) cohort. Nephrol Dial Transplant.

[18] Levey AS, Bosch JP, Lewis JB, Greene T, Rogers N, Roth D (1999). A more accurate method to estimate glomerular filtration rate from serum creatinine: a new prediction equation. Modification of Diet in Renal Disease Study Group. Ann Intern Med.

[19] American Diabetes Association (2010). Diagnosis and classification of diabetes mellitus. Diabetes Care.

[20] Levey AS, Stevens LA, Schmid CH, Zhang YL, Castro AF, Feldman HI (2009). A new equation to estimate glomerular filtration rate. Ann Intern Med.

[21] KDOQI Clinical Practice Guideline for Diabetes and CKD (2012). 2012 Update. Am J Kidney Dis.

[22] Bundesärztekammer (BÄK), Arbeitsgemeinschaft der Wissenschaftlichen Medizinischen Fachgesellschaften (AWMF). Nationale Versorgungsleitlinie Nierenerkrankungen bei Diabetes im Erwachsenenalter, 1. Auflage 2010.

[23] Ott P, Benke I, Stelzer J, Kohler C, Hanefeld M (2009). “Diabetes in Germany” (DIG) study. A prospective 4-year-follow-up study on the quality of treatment for type 2 diabetes in daily practice. Dtsch Med Wochenschr.

[24] Stone MA, Charpentier G, Doggen K, Kuss O, Lindblad U, Kellner C (2013). Quality of care of people with type 2 diabetes in eight European countries: findings from the Guideline Adherence to Enhance Care (GUIDANCE) study. Diabetes Care.

[25] Shurraw S, Hemmelgarn B, Lin M, Majumdar SR, Klarenbach S, Manns B (2011). Association between glycemic control and adverse outcomes in people with diabetes mellitus and chronic kidney disease: a population-based cohort study. Arch Intern Med.

[26] Zoungas S, Patel A, Chalmers J, de Galan BE, Li Q, Billot L (2010). Severe hypoglycemia and risks of vascular events and death. N Engl J Med.

[27] Hemmingsen B, Lund SS, Gluud C, Vaag A, Almdal T, Hemmingsen C (2013). Targeting intensive glycaemic control versus targeting conventional glycaemic control for type 2 diabetes mellitus. Cochrane Database Syst Rev.

[28] Perkovic V, Heerspink HL, Chalmers J, Woodward M, Jun M, Li Q (2013). Intensive glucose control improves kidney outcomes in patients with type 2 diabetes. Kidney Int.

[29] Patel A, MacMahon S, Chalmers J, Neal B, Billot L, Woodward M (2008). Intensive blood glucose control and vascular outcomes in patients with type 2 diabetes. N Engl J Med.

[30] Inaba M, Okuno S, Kumeda Y, Yamada S, Imanishi Y, Tabata T (2007). Glycated albumin is a better glycemic indicator than glycated hemoglobin values in hemodialysis patients with diabetes: effect of anemia and erythropoietin injection. J Am Soc Nephrol.

[31] Wolf G, Muller N, Hunger-Battefeld W, Kloos C, Muller UA (2008). Hemoglobin concentrations are closely linked to renal function in patients with type 1 or 2 diabetes mellitus. Kidney Blood Press Res.

[32] Muller N, Heller T, Freitag MH, Gerste B, Haupt CM, Wolf G (2015). Healthcare utilization of people with type 2 diabetes in Germany: an analysis based on health insurance data. Diabet Med.

[33] Rafaniello C, Arcoraci V, Ferrajolo C, Sportiello L, Sullo MG, Giorgianni F (2015). Trends in the prescription of antidiabetic medications from 2009 to 2012 in a general practice of Southern Italy: a population-based study. Diabetes Res Clin Pract.

[34] Yurgin N, Secnik K, Lage MJ (2007). Antidiabetic prescriptions and glycemic control in German patients with type 2 diabetes mellitus: a retrospective database study. Clin Ther.

[35] Baviera M, Monesi L, Marzona I, Avanzini F, Monesi G, Nobili A (2011). Trends in drug prescriptions to diabetic patients from 2000 to 2008 in Italy’s Lombardy Region: a large population-based study. Diabetes Res Clin Pract.

[36] Currie CJ, Peters JR, Tynan A, Evans M, Heine RJ, Bracco OL (2010). Survival as a function of HbA(1c) in people with type 2 diabetes: a retrospective cohort study. Lancet.

[37] Flory JH, Hennessy S (2015). Metformin use reduction in mild to moderate renal impairment: possible inappropriate curbing of use based on food and drug administration contraindications. JAMA Intern Med.

[38] Lu WR, Defilippi J, Braun A (2013). Unleash metformin: reconsideration of the contraindication in patients with renal impairment. Ann Pharmacother.

[39] National Institute for Health and Care Excellence (2009). Type 2 diabetes: national clinical guideline for management in primary and secondary care (update).

[40] Inzucchi SE, Bergenstal RM, Buse JB, Diamant M, Ferrannini E, Nauck M (2012). Management of hyperglycemia in type 2 diabetes: a patient-centered approach: position statement of the American Diabetes Association (ADA) and the European Association for the Study of Diabetes (EASD). Diabetes Care.

[41] Speeckaert M, Van Biesen W, Delanghe J, Slingerland R, Wiecek A, Heaf J (2014). Are there better alternatives than haemoglobin A1c to estimate glycaemic control in the chronic kidney disease population?. Nephrol Dial Transplant.

[42] Szymezak J, Lavalard E, Martin M, Leroy N, Gillery P (2009). Carbamylated hemoglobin remains a critical issue in HbA1c measurements. Clin Chem Lab Med.

