# Supplementary figures and images for: Glycaemic control and antidiabetic therapy in patients with diabetes mellitus and chronic kidney disease – cross-sectional data from the German Chronic Kidney Disease (GCKD) cohort
Source: BMC Nephrol. 2016 Jun 11;17:59. doi: 10.1186/s12882-016-0273-z (PMC4902996; doi:10.1186/s12882-016-0273-z)

## Slide 1
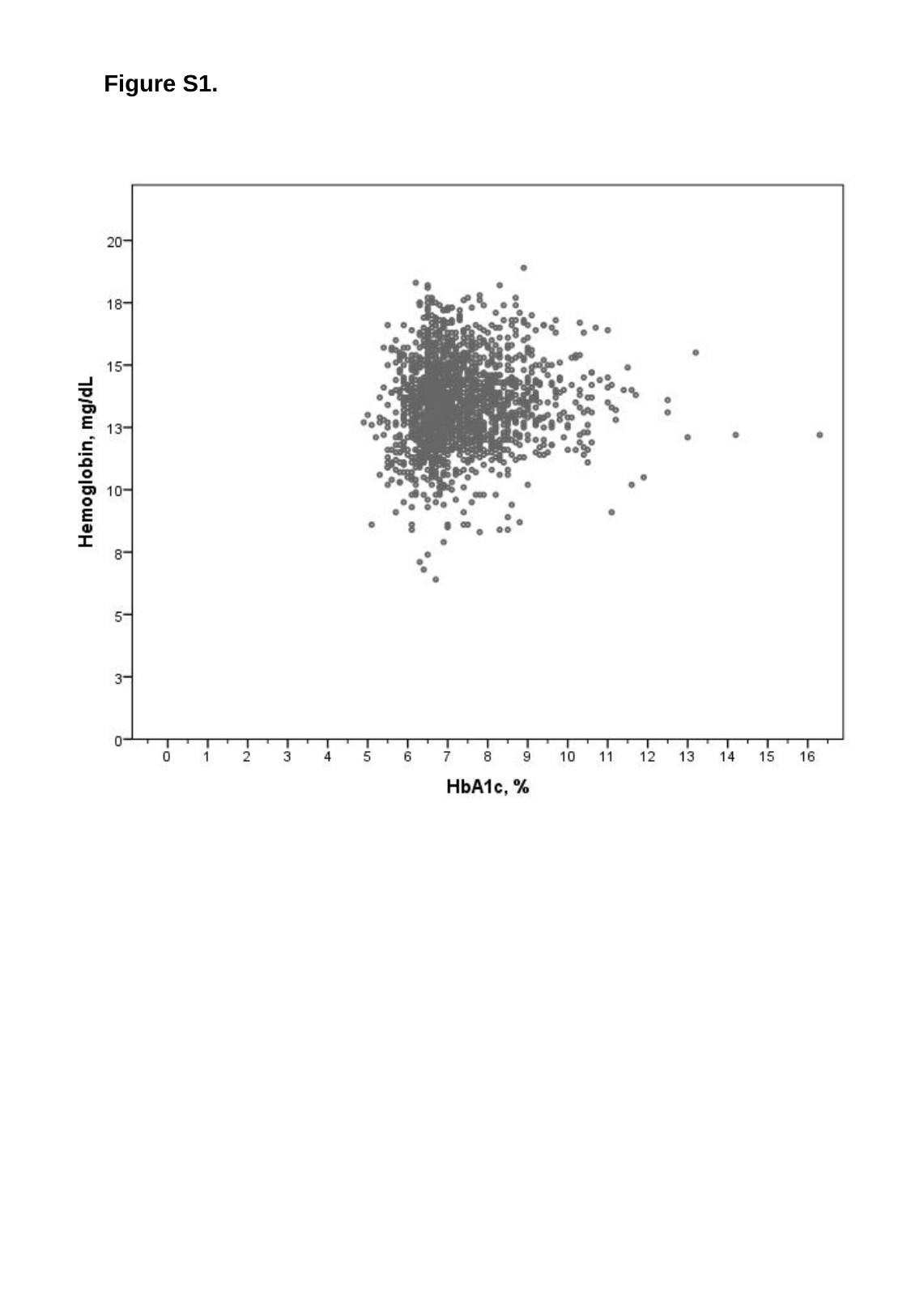

Figure S1.

Supplement: Additional file 1: Figure S1. — Association between hemoglobin and hemoglobin A1C (HbA1C) values in 1775 patients having diabetes mellitus and stage 3 chronic kidney disease and/or overt proteinuria (r = 0.082, p < 0.001), for conversion of hemoglobin into SI units (mmol/L): multiply with 0.62. (PPT 81 kb) [file 12882_2016_273_MOESM1_ESM.ppt]

## Slide 1
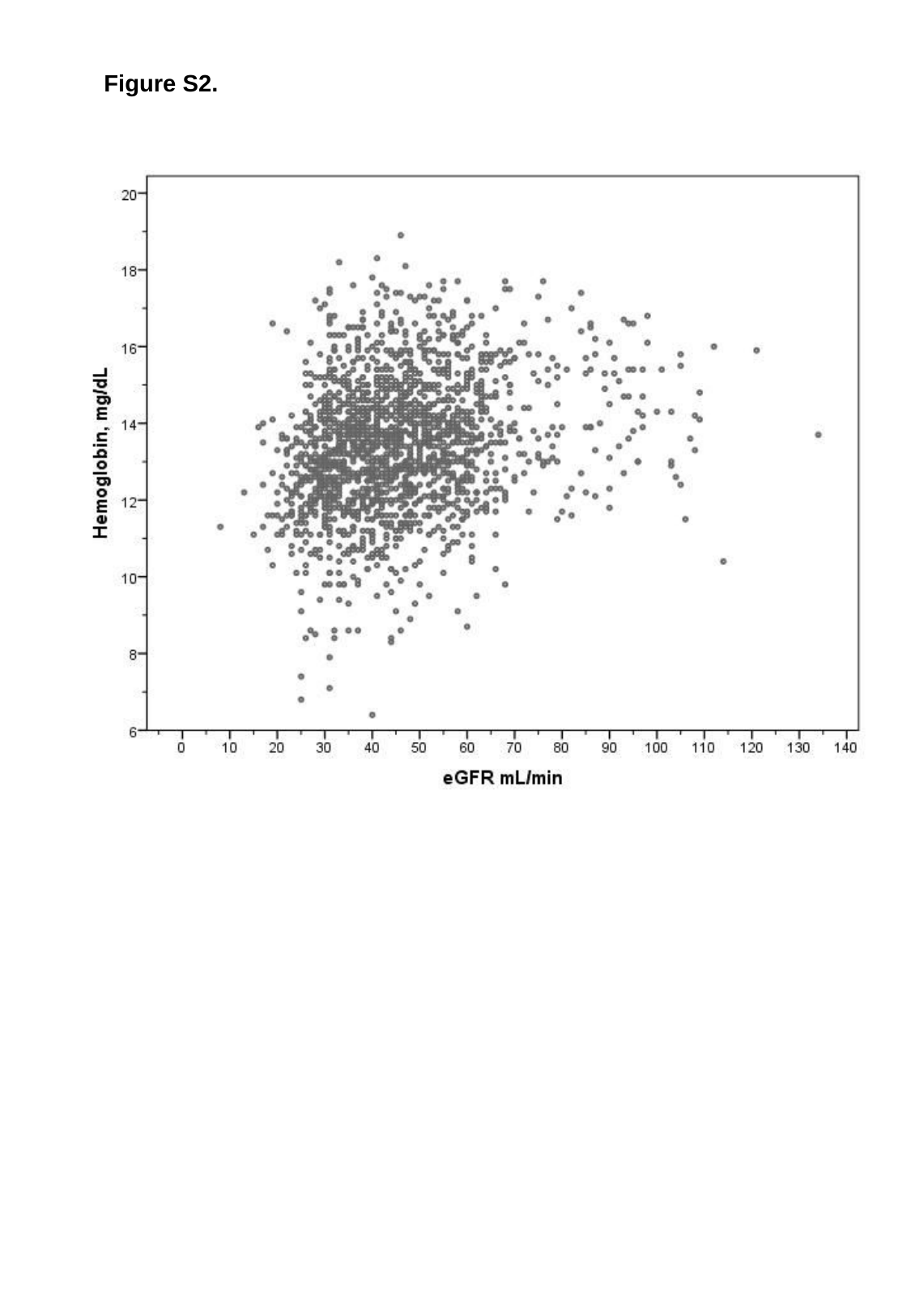

Figure S2.

Supplement: Additional file 2: Figure S2. — Association between hemoglobin and estimated glomerular filtration rate (eGFR) in 1759 patients having diabetes mellitus and stage 3 chronic kidney disease and/or overt proteinuria (r = 0.247, p < 0.0001). (PPT 103 kb) [file 12882_2016_273_MOESM2_ESM.ppt]
