# Supplementary material for: Glycaemic control and antidiabetic therapy in patients with diabetes mellitus and chronic kidney disease – cross-sectional data from the German Chronic Kidney Disease (GCKD) cohort
Source: BMC Nephrol. 2016 Jun 11;17:59. doi: 10.1186/s12882-016-0273-z (PMC4902996; doi:10.1186/s12882-016-0273-z)
Supplement: Additional file 3: Table S1. — Correlates of median HbA1C levels >7.0 % (53 mmol/mol) according to logistic regression analysis (entire model). (DOCX 24 kb) [file 12882_2016_273_MOESM3_ESM.docx]

**Table S1 Correlates of median HbA1C levels >7.0% (53 mmol/mol) according to logistic regression analysis (entire model)**

| ***Indicators ^a, b, c^*** | ***Regression coefficient*** | ***Standard error*** | ***Odds ratio*** | ***95% confidence interval*** | ***P-value*** |
| --- | --- | --- | --- | --- | --- |
| **Age**  **(per 1 year increase)** | -0.00938 | 0.00702 | 0.991 | 0.977 – 1.004 | 0.18 |
| **Gender**  **(male vs. female)** | 0.0257 | 0.1238 | 1.026 | 0.805 – 1.308 | 0.84 |
| **Duration of CKD ^a^**  ≥ 5 years | 0.00576 | 0.2172 | 1.006 | 0.657 – 1.540 | 0.98 |
| 3 - < 5 years | 0.1356 | 0.2427 | 1.145 | 0.712 – 1.843 | 0.58 |
| 1 - < 3 years | -0.1099 | 0.2315 | 0.896 | 0.569 – 1.410 | 0.63 |
| **Physical activity** ^b^  1-2 times a week | 0.1625 | 0.1715 | 1.176 | 0.841 – 1.646 | 0.34 |
| 3-5 times a week | 0.1184 | 0.1668 | 1.126 | 0.812 – 1.561 | 0.48 |
| More than 5 times a week | 0.1991 | 0.1649 | 1.220 | 0.883 – 1.686 | 0.23 |
| **eGFR, *mL/min/1.73m²***  **(per 1 mL/min increase)** | -0.00466 | 0.00405 | 0.995 | 0.987 – 1.003 | 0.25 |
| **Body mass index**  **(per 1 kg/m² increase)** | 0.0390 | 0.00964 | 1.040 | 1.020 – 1.060 | < 0.0001 |
| **Hemoglobin**  **(per 1 g/dL increase) ^d^** | 0.1080 | 0.0356 | 1.114 | 1.039 – 1.195 | 0.0024 |
| **C-reactive protein**  **(per 1 mg/L increase)** | 0.00719 | 0.00693 | 1.007 | 0.994 – 1.021 | 0.30 |
| ***antidiabetic treatment strategies ^c^*** | | | | | |
| **Oral anti-diabetic drugs alone, *any* (n=426)** | 0.2554 | 0.1585 | 1.291 | 0.946 – 1.761 | 0.11 |
| **Oral anti-diabetic drugs plus insulin (n=141)** | 1.4503 | 0.2185 | 4.264 | 2.779 – 6.544 | < 0.0001 |
| **Insulin alone (n=699)** | 1.7256 | 0.1439 | 5.616 | 4.236 – 7.445 | < 0.0001 |

N=266 observations were excluded from the analysis due to combinations that were used less frequently and missing values.

^a^ CKD duration of < 1 year was used as the reference category

^b^ physical activity less than once a week was used as the reference category

^c^ dietary treatment was used as the reference category for any group of antidiabetic therapy

^d^ for conversion into SI units (mmol/L): multiply with 0.62
