# Supplementary material for: Glycaemic control and antidiabetic therapy in patients with diabetes mellitus and chronic kidney disease – cross-sectional data from the German Chronic Kidney Disease (GCKD) cohort
Source: BMC Nephrol. 2016 Jun 11;17:59. doi: 10.1186/s12882-016-0273-z (PMC4902996; doi:10.1186/s12882-016-0273-z)
Supplement: Additional file 4: Table S2. — Correlates of median HbA1C levels >7.0 % (53 mmol/mol) including the most frequently used antidiabetic therapies according to stepwise logistic regression analysis (final model). (DOCX 22 kb) [file 12882_2016_273_MOESM4_ESM.docx]

**Table S2 Correlates of median HbA1C levels >7.0% (53 mmol/mol) including the most frequently used antidiabetic therapies according to stepwise logistic regression analysis (final model)**

| ***Indicators ^a, b^*** | ***Regression coefficient ^a^*** | ***Standard error ^a^*** | ***Odds ratio ^a^*** | ***95% confidence interval ^a^*** | ***P-value ^a^*** |
| --- | --- | --- | --- | --- | --- |
| **Body mass index**  **(per 1 kg/m² increase)** | 0.0390 | 0.0093 | 1.040 | 1.021 – 1.059 | < 0.0001 |
| **Hemoglobin**  **(per 1 g/dL increase) ^c^** | 0.1102 | 0.0326 | 1.116 | 1.047 – 1.190 | 0.0007 |
| ***antidiabetic medication ^b^*** | | | | | |
| DPP-4 inhibitors (n=46) | -0.8350 | 0.3312 | 0.864 | 0.418 – 1.790 | 0.0117 |
| DPP-4 inhibitors  + Insulin (n=27) | 0.5264 | 0.3836 | 3.373 | 1.456 – 7.811 | 0.17 |
| Glinides (n=59) | -0.7969 | 0.2951 | 0.898 | 0.470 – 1.717 | 0.0069 |
| Insulin (n=699) | 1.0416 | 0.1185 | 5.646 | 4.267 – 7.470 | < 0.0001 |
| Metformin (n=123) | -0.8002 | 0.2140 | 0.895 | 0.555 – 1.443 | 0.0002 |
| Metformin +  DPP-4 inhibitors (n=37) | -0.7195 | 0.3534 | 0.970 | 0.448 – 2.102 | 0.0417 |
| Metformin + Insulin (n=76) | 0.5524 | 0.2379 | 3.462 | 2.039 – 5.876 | 0.0203 |
| Metformin  + Sulfonylureas (n=38) | 0.5627 | 0.3157 | 3.497 | 1.748 – 6.998 | 0.07 |
| Sulfonylureas (n=123) | -0.1974 | 0.1961 | 1.636 | 1.054 – 2.537 | 0.31 |
| Sulfonylureas + Insulin (n=38) | 1.3553 | 0.3615 | 7.726 | 3.503 – 17.043 | 0.0002 |

N=266 observations were deleted due to missing values

^a^ according to the final model; age, gender, BMI, duration of CKD, physical activity, eGFR, hemoglobin, CRP,

and antidiabetic medication were used as variables in the initial model

^b^ dietary treatment was used as the reference category for any group of antidiabetic therapy

^c^ for conversion into SI units (mmol/L): multiply with 0.62
