# Supplementary material for: Glycaemic control and antidiabetic therapy in patients with diabetes mellitus and chronic kidney disease – cross-sectional data from the German Chronic Kidney Disease (GCKD) cohort
Source: BMC Nephrol. 2016 Jun 11;17:59. doi: 10.1186/s12882-016-0273-z (PMC4902996; doi:10.1186/s12882-016-0273-z)
Supplement: Additional file 5: Table S3. — Correlates of median HbA1C levels >7.0 % (53 mmol/mol) including the most frequently used antidiabetic therapies according to logistic regression analysis (entire model). (DOCX 24 kb) [file 12882_2016_273_MOESM5_ESM.docx]

**Table S3 Correlates of median HbA1C levels >7.0% (53 mmol/mol) including the most frequently used antidiabetic therapies according to logistic regression analysis (entire model)**

| ***Indicators ^a, b, c^*** | ***Regression coefficient*** | ***Standard error*** | ***Odds ratio*** | ***95% confidence interval*** | ***P-value*** |
| --- | --- | --- | --- | --- | --- |
| **Age**  **(per 1 year increase)** | -0.0109 | 0.00709 | 0.989 | 0.975 – 1.003 | 0.12 |
| **Gender**  **(male vs. female)** | 0.00283 | 0.0626 | 1.006 | 0.787 – 1.285 | 0.96 |
| **Duration of CKD ^a^**  ≥ 5 years | -0.0191 | 0.1210 | 1.002 | 0.652 – 1.542 | 0.87 |
| 3 - < 5 years | 0.1265 | 0.1531 | 1.160 | 0.717 – 1.875 | 0.41 |
| 1 - < 3 years | -0.1549 | 0.1397 | 0.875 | 0.553 – 1.385 | 0.27 |
| **Physical activity** ^b^  1-2 times a week | 0.1564 | 0.1461 | 1.161 | 0.827 – 1.628 | 0.28 |
| 3-5 times a week | 0.1322 | 0.1427 | 1.133 | 0.815 – 1.575 | 0.35 |
| More than 5 times a week | 0.2017 | 0.1411 | 1.214 | 0.877 – 1.682 | 0.15 |
| **eGFR, *mL/min/1.73m²***  **(per 1 mL/min increase)** | -0.00433 | 0.00416 | 0.996 | 0.988 – 1.004 | 0.30 |
| **Body mass index**  **(per 1 kg/m² increase)** | 0.0406 | 0.00977 | 1.041 | 1.022 – 1.062 | < 0.0001 |
| **Hemoglobin**  **(per 1 g/dL increase) ^d^** | 0.1118 | 0.0360 | 1.118 | 1.042 – 1.200 | 0.0019 |
| **C-reactive protein**  **(per 1 mg/L increase)** | 0.00602 | 0.00695 | 1.006 | 0.992 – 1.020 | 0.39 |
| ***antidiabetic medication ^c^*** | | | | | |
| DPP-4 inhibitors (n=46) | -0.8593 | 0.3344 | 0.859 | 0.412 – 1.790 | 0.0102 |
| DPP-4 inhibitors  + Insulin (n=27) | 0.4804 | 0.3879 | 3.280 | 1.404 – 7.659 | 0.22 |
| Glinides (n=59) | -0.8334 | 0.2976 | 0.882 | 0.459 – 1.692 | 0.0051 |
| Insulin (n=699) | 1.0208 | 0.1209 | 5.631 | 4.245 – 7.468 | < 0.0001 |
| Metformin (n=123) | -0.7686 | 0.2183 | 0.941 | 0.577 – 1.533 | 0.0004 |
| Metformin +  DPP-4 inhibitors (n=37) | -0.6383 | 0.3571 | 1.072 | 0.491 – 2.340 | 0.07 |
| Metformin + Insulin (n=76) | 0.5623 | 0.2422 | 3.560 | 2.077 – 6.101 | 0.0202 |
| Metformin  + Sulfonylureas (n=38) | 0.5650 | 0.3204 | 3.569 | 1.763 – 7.227 | 0.08 |
| Sulfonylureas (n=123) | -0.1547 | 0.1987 | 1.738 | 1.115 – 2.709 | 0.44 |
| Sulfonylureas + Insulin (n=38) | 1.3332 | 0.3654 | 7.696 | 3.460 – 17.117 | 0.0003 |

N=266 observations were deleted due to missing values

^a^ CKD duration of < 1 year was used as the reference category

^b^ physical activity less than once a week was used as the reference category

^c^ dietary treatment was used as the reference category for any group of antidiabetic therapy

^d^ for conversion into SI units (mmol/L): multiply with 0.62
